# Reducing heat risks for the elderly by rescheduling healthcare appointments in Germany

**DOI:** 10.1016/j.pmedr.2025.103309

**Published:** 2025-11-11

**Authors:** Philipp Sprengholz, Robert W. Bruckmann

**Affiliations:** a Institute of Psychology, University of Bamberg, Bamberg, Germany; b Institute for Planetary Health Behavior, University of Erfurt, Erfurt, Germany; c Health Communication Working Group, Bernhard Nocht Institute for Tropical Medicine, Hamburg, Germany

**Keywords:** Heat, Health care, Consultation hours, Patients, Elderly

## Abstract

**Objective:**

Intensifying heatwaves pose severe health risks for elderly individuals, particularly during healthcare appointments scheduled in hot periods. We aimed to investigate if shifting consultation times or rescheduling elderly patients may mitigate heat-related risks and if healthcare providers and patients are open to do so.

**Methods:**

We analyzed 17,619,866 consultation hours from 90,977 German healthcare providers during summer 2024, linking them with weather data to investigate heat-related risks. Simulations tested the effects of advancing, postponing, and mixed scheduling strategies. Additionally, surveys with 459 healthcare providers and 259 elderly patients assessed rescheduling practices, attitudes, and acceptance.

**Results:**

Analyses revealed that 15 % of consultations occurred under caution-level and 1 % under extreme caution-level heat conditions, with risk peaking in afternoon hours. Simulations showed that advancing morning and postponing afternoon consultations significantly reduced unsafe heat exposure. While 59 % of practices could theoretically accommodate all elderly patients before 11 am, few were doing so. Provider-initiated rescheduling was rare but more likely among those recognizing heat risks. Patient surveys revealed strong acceptance of rescheduling, particularly to morning hours.

**Conclusions:**

Rescheduling healthcare appointments offers a feasible strategy to reduce heat risks for elderly patients. Increasing provider awareness may foster proactive rescheduling practices, enhancing patient protection during heatwaves.

## Highlights

• Some healthcare consultations occur under heat-risk conditions.
• Heat risks peak in afternoon; advancing hours reduces unsafe exposure.
• Many elderly patients open to morning and evening rescheduling.
• Providers rarely initiate rescheduling, despite clear health benefits.

## 1 Introduction

Heat-related illnesses and mortality increase with the rising frequency, intensity, and duration of heatwaves across the globe (Perkins-Kirkpatrick and Lewis, 2020). In Europe alone, more than 47,000 people died in 2023 owing to high temperatures (Gallo et al., 2024). Heat risks are particularly high for the elderly (Ballester et al., 2023), attributable to physiological changes, comorbidities and their treatments, and lack of adaptability (Hansen et al., 2011; Wee et al., 2023). Previous evidence suggests that older individuals often underestimate heat risks (Oberai et al., 2024). While educational efforts can help to increase awareness and protective behavior (Hansen et al., 2011), experts have been emphasizing structural changes that reduce heat exposure, such as making flats more heat-resistant or improving the shading of outdoor areas (Jay et al., 2021; Kenny et al., 2024). Heat risks can also be reduced by aiding individuals to shift specific activities to cooler daytime hours. For instance, older individuals interact with healthcare providers regularly (Nielsen and Andersen, 2025), and in many cases, these interactions take place during hot days. Travel to and from a clinic is likely to expose patients to heat, and many individuals may ignore adequate hydration (Kenney and Chiu, 2001) and appropriate clothing for their travel and the time in the waiting room. To reduce heat-related health risks, healthcare providers could schedule alternative appointments for elderly patients at a cooler time of day. This may also improve heat protection in general; by asking patients if an appointment should be rescheduled, they may become more aware of the heat and adapt their behavior.

In this article, we investigate the heat risk associated with healthcare appointments by analyzing the weather data associated with consultation times of 90,977 medical and psychotherapeutic practices in the summer of 2024 in Germany. We aim to demonstrate how shifting consultation hours and rescheduling elderly patients' appointments can reduce this risk. By integrating data from practice and patient surveys, we further examine if and under which circumstances healthcare providers offer rescheduled appointments to elderly patients, and which rescheduling windows are commonly accepted by the patient population. The findings may serve to understand the benefits and barriers of rescheduling appointments during heatwaves and may inform recommendations and policies that promote rescheduling activities among healthcare providers.

## 2 Methods

Different data sources were used to investigate the potential of shifting and rescheduling healthcare consultations: (a) official consultation hours, (b) a healthcare provider survey, and (c) a survey of elderly patients.

### 2.1 Consultation and weather data

Summer consultation hours of German medical and psychotherapeutic practices were sourced from the nationwide consultation hours registry of the National Association of Statutory Health Insurance Physicians. A consultation hour was defined as an hour in which a healthcare provider is open for consultation for at least 30 min. As consultation hours were only available for 30 days in advance and the collection process took about 3 days, complete data was collected for two periods, 9th July to 4th August and 8th August to 30th August 2024. In total, *N* = 17,619,866 consultation hours of 90,977 healthcare providers were sourced. For each provider, the email address (if available) and location were collected. This allowed us to invite the providers to a survey (see next section) and map weather data to each consultation hour. Specifically, air temperature and relative humidity recorded at the middle of each consultation hour were integrated from the Integrated Forecasting System (IFS) model of the European Centre for Medium-Range Weather Forecasts (ECMWF, 2024). For each consultation hour, a heat index was calculated from air temperature and relative humidity and classified as *safe* (values below 27), *caution* (values from 27 to 32, where prolonged exposure or physical activity can result in fatigue), or *extreme caution* (values from 33 to 40, where health impacts can extend to heat strokes, cramps and heat exhaustion). The calculation was based on an algorithm proposed by the US National Weather Service (2022) and implemented in the *weathermetrics* package (version 1.2.2) for R; see Anderson et al. (2013) for a detailed specification with formulas. Whereas even higher heat indexes with worse health impacts (values above 40) are possible, they were not observed in the data.

### 2.2 Healthcare provider survey

All German healthcare providers that were listed in the nationwide consultation hours registry of the National Association of Statutory Health Insurance Physicians with an email address (*n* = 26,173) were invited to participate in an online survey in September 2024. In total, 1125 healthcare providers participated, but only *N* = 459 indicated that they were open and treating individuals above 65 years in July and August 2024, constituting the final analytical sample. The sample size was deemed sufficient for detecting medium-sized differences (*d* = 0.5) in perceptions and attitudes between providers that did versus did not offer rescheduling; according to an a-priori power analysis with G*Power 3.1 (Faul et al., 2009) data from at least 278 providers had to be collected (when assuming that only about 10 % of providers may offer rescheduling, α = 0.05, 1-β = 0.80).

At the beginning of the survey, healthcare providers were asked to estimate how many patients who had an appointment in the last two summer months (July and August) were above 65 years (0–100 %). They further estimated how many of these patients had an appointment before 11 am (0–100 %). Afterward, healthcare providers were asked if they had been contacted in the last two months by patients who explicitly intended to postpone their appointment due to the high temperatures (yes/no) and if they had contacted patients for the same reason (yes/no). At the end of the survey, agreement with the statements “In my opinion, heat is a relevant health risk for people over the age of 65” and “I see it as my duty to point out the health risks of heat to people who are being treated by us and to inform them about protective behavior” was assessed using a 7-point scale from “strongly disagree” to “strongly agree”. The items were designed specifically for this study.

### 2.3 Patient survey

A total of 483 German adults over 60 years of age self-enrolled in an incentivized lottery-based panel to repeatedly answer online surveys on heat perceptions and actions between June and September 2024. In July 2024, *N* = 259 individuals completed a short survey on the rescheduling of healthcare appointments during heatwaves. Respondents ranged in age from 60 to 86 years (*M* = 69.53 years, *SD* = 4.15 years); 58 % were female, and 42 % were male.

Participants were asked to imagine that their general practitioner called the day before an appointment. The appointment was scheduled for the afternoon; the temperature for that afternoon was forecast to be high, so the practice suggests rescheduling the appointment. In eight tasks, participants had to decide if they wanted to keep the original appointment or if they would instead agree to shift it to 5, 6, 7, or 8 am, or to 6, 7, 8, or 9 pm. Some of these time slots clearly deviate from normal working hours but we wanted to investigate patients' interest in shifting to early or late hours beyond current opening patterns.

### 2.4 Ethics and consent

The surveys followed the guidelines of the German Psychological Association. Ethical clearance was obtained from the University of Bamberg's institutional review board (#2024–04/21), and all participants provided informed consent for their data to be used and shared for scientific purposes without identity disclosure. Participation in the surveys was unpaid. However, taking part in the elderly panel was incentivized with a lottery.

### 2.5 Statistical analysis

We investigated the proportion of safe and unsafe consultation hours at different times of the day, and how this proportion changed when consultation hours were shifted to earlier or later times. Descriptive analyses were used to examine healthcare provider and patient survey data. Specifically, we investigated how many elderly patients could potentially be treated during safe consultation hours and how many providers offered rescheduling services. *t*-tests were conducted to determine whether rescheduling was more likely among providers who were aware of heat risks and recognised their responsibility to protect patients from heat. Responses from the patient survey were used to calculate the proportion of patients who agreed to reschedule an appointment to morning or evening hours. R (version 4.3.1) was used for all analyses.

## 3 Results

### 3.1 Heat during consultation hours

**Fig. 1:**
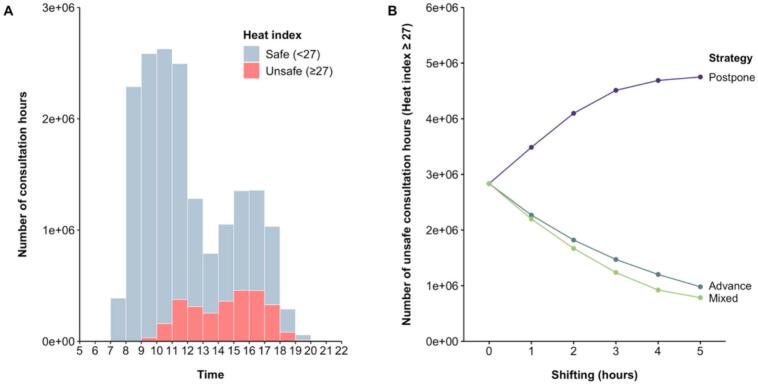
**Heat risk associated with (shifting) consultation hours.***Footnote:* Panel A shows the distribution of 17,619,866 consultation hours of 90,977 German healthcare providers offered between 9th July and 4th August and between 8th and 30th August 2024 (50 days) by hour of day and associated heat index. Panel B indicates how the total number of unsafe hours (defined by a heat index of 27 or higher) changes when consultation times are postponed (shifted to later times but not later than 10 pm), advanced (shifted to earlier times but not earlier than 5 am), or shifted with a mixed strategy (advanced before and postponed after 2 pm) by one or multiple hours.

In total, 17,619,866 consultation hours with associated weather data from July and August 2024 were analyzed. As shown in Fig. 1A, most of them (95.7 %) ranged between 8 am and 6 pm, with a drop in the numbers around noon. In general, heat indexes could be considered safe for most consultation hours, while 15 % warranted caution and 1 % extreme caution. Importantly, the share of consultation hours with increased heat risk peaked post-noon. For example, between 8 and 9 am, less than 1 % of consultation hours were unsafe, while the share rose to 34 % between 2 and 3 pm.

### 3.2 Effects of shifting consultation hours

Shifting consultation hours to earlier or later times could help to reduce the heat burden of healthcare appointments on hot days. We investigated the effects of three shifting strategies on the number of unsafe consultation hours: advancing (shifting all hours to earlier times but not earlier than 5 am), postponing (shifting all hours to later times but not later than 10 pm) and a mixed approach (advancing and postponing hours before and after 2 pm, respectively). As shown in Fig. 1B, the number of unsafe consultation hours increased with postponing, but decreased with advancing, and especially with the mixed approach. The positive effects of advancing and the mixed approach increased as the hours shifted further. For example, when consultations were advanced (shifted with the mixed approach) by one hour, this led to a reduction of unsafe hours by 20 % (22 %), while shifting by three hours led to a reduction by 48 % (56 %). In summary, this simulation shows that advancing morning consultations can reduce heat-related health risks, and further benefits can be achieved by postponing afternoon appointments in a mixed strategy.

### 3.3 Rescheduling appointments of elderly patients in theory and practice

**Fig. 2:**
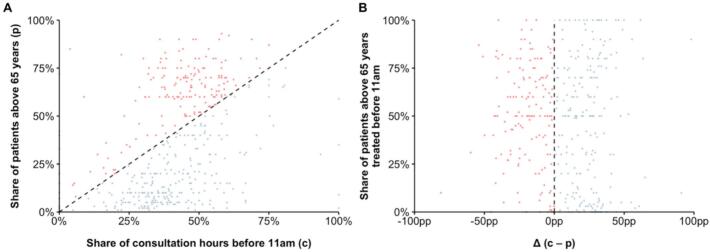
**Shares of morning consultation hours, elderly patients, and those treated during morning consultation hours.***Footnote:* Panel A contrasts the share of actual consultation hours before 11 am with the share of patients above 65 years reported by 459 German healthcare providers for July and August 2024 in an online survey conducted in September 2024 (each provider visualized as a dot). When assuming that consultation durations are similar for all patients, 59 % of healthcare providers could accommodate all patients above 65 years before 11 am (blue dots, right of the dashed line). Panel B indicates that this was rarely the case, as most providers reported to only treat a share of these patients before 11 am. (For interpretation of the references to colour in this figure legend, the reader is referred to the web version of this article.)

Shifting consultation hours reduces heat risks for all patients but may not be practical or feasible for several healthcare providers. Rescheduling patients within the current consultation times could be an alternative to protect the most vulnerable. For instance, elderly individuals could be offered consultations in the morning hours, whereas relatively less vulnerable population groups could be received at later (hotter) times. The potential and use of this strategy were assessed in the healthcare provider survey. Among providers that had treated patients above 65 years in July and August 2024, the share of these patients ranged from 1 % to 93 % (*M* = 35 %, *SD* = 27 pp). As shown in Fig. 2A, we could compare this share with the percentage of morning consultation hours (i.e., hours before 11 am in relation to all hours) for each practice. When assuming that consultation durations are similar for all patients, 59 % of healthcare providers could (in principle) accommodate all patients above 65 years before 11 am. This contrasts with reality as these providers reported that on average only 45 % (*SD* = 34 pp) of their patients above 65 years had such early appointments, putting large parts of this vulnerable population at unnecessary risk (Fig. 2B).

Further survey results underline the untapped potential of rescheduling appointments within existing consultation hours. About a fifth of healthcare providers (19 %) reported that they had been contacted by patients asking to reschedule an appointment because of the excess heat. However, provider-initiated rescheduling prior to or on hot days was generally low (7 %). Interestingly, rescheduling requests by patients and providers were correlated (*r* = 0.15, *p* = 0.002). Further analyses revealed that providers' risk perception and self-image related to their rescheduling activities. Specifically, providers who rescheduled appointments were more likely to think that heat poses a health risk for people above 65 years (*M* = 6.19, *SD* = 1.01) compared to those who did not (*M* = 5.72, *SD* = 1.41, Welch's *t*(39.74) = 2.42, *p* = 0.020, *d* = 0.38). Furthermore, rescheduling providers were more convinced that it is their duty to point out the health risks of heat and inform about protective behavior (*M* = 5.42, *SD* = 1.54) compared to providers who did not offer to reschedule appointments (*M* = 4.22, *SD* = 1.95, Welch's *t*(37.78) = 4.08, *p* < 0.001, *d* = 0.68).

### 3.4 Patient support for rescheduling appointments

Rescheduling healthcare appointments may only be feasible if it is positively received and accepted by patients. To that end, the patient survey assessed whether individuals above 65 years would accept the shifting of a noon appointment with their general practitioner to morning or evening hours on a hot day. Most patients accepted rescheduling to 8 am (68 %), while acceptance decreased for earlier times (37 % for 7 am, 17 % for 6 am, and 11 % for 5 am). A similar pattern could be observed for evening times; while 47 % accepted rescheduling to 6 pm, acceptance decreased for later times (41 % for 7 pm, 29 % for 8 pm, and 17 % for 9 pm). However, only 12 % answered that they would not accept the rescheduling of their appointment to any of these morning and evening times.

## 4 Discussion

This study investigated heat risks associated with healthcare appointments and investigated how shifting consultation hours and rescheduling vulnerable patients could mitigate them.

Analyses of German consultation records and weather data showed that considerable shares of healthcare consultations occur during severe heat. Thus, patients may experience high temperatures when travelling to and from the practice or spending extended time in a waiting room without air condition. This poses a particular risk to older patients due to physiological changes such as reduced thermoregulation and blunted thirst perception (often resulting in inadequate hydration and inappropriate clothing, Kenney and Chiu, 2001), as well as comorbidities and medications that can increase heat sensitivity.

Simulations showed that heat-related risks can be reduced by advancing morning and postponing afternoon appointments. While this would benefit all patients, an easier alternative to shifting consultation times could be to reschedule vulnerable populations within the current opening hours. For example, instead of shifting consultation times from 9 to 12 am to 6 to 9 am, the heat burden could also be reduced by ensuring that vulnerable patients are treated in the earlier hours, i.e., at 9 instead of 11 am. Respective analyses suggested that a majority of practices could see all their elderly patients before 11 am, but only a few reported doing so. The survey of healthcare providers further indicated that they rarely approached patients with rescheduling proposals. Importantly, rescheduling was more common when providers considered heat a health risk and when they understood informing about and acting on heat protection as an integral part of their work. This calls for educational interventions that (a) inform healthcare providers about the adverse consequences of heat and (b) suggest appropriate interventions for mitigating risks in their own practice, such as rescheduling appointments of vulnerable populations. Although not focused on in this study, asking patients to reschedule an appointment could not only reduce heat risks but also act as a threat signal, thereby increasing awareness and protective behavior. Some support for this positive effect comes from the observation that patients were more likely to request the rescheduling of appointments from providers who did so too.

Some limitations should be noted. The findings relate to the German context; consultation times, weather, and the behavior of healthcare providers may differ in other regions. Furthermore, we chose the heat index as a rather conservative measure of heat-related health risk. The calculation of thermal comfort indexes and risk levels has been subject to ongoing discussions, and calculations may deviate when additional weather and patient data (e.g., wind speed, metabolic rate, solar radiation) are considered (Lu and Romps, 2023). The weather model used in our analysis has high precision overall, rasterizing to a 9-km resolution, but may miss micro-scale thermodynamics in dense urban areas where practitioners are often located. The heat index may therefore be underestimated.

The survey results may not be representative of all healthcare providers or patients. It is likely that only healthcare providers interested in heat protection participated, meaning that the actual proportion of providers offering rescheduling services may be even lower. Similarly, the surveyed patients were enrolled in a panel study on heat perception and behavior. As they may have been more aware of heat risks than other elderly individuals, the general population's willingness to reschedule appointments to early or late hours may be lower. Future surveys with representative samples could help review our results and identify potential biases.

While rescheduling can help to protect vulnerable populations, it may not always be feasible. The practice only makes sense for planned routine visits, and it requires some flexibility on both the patient and provider side. While our data suggests that many patients are open to rescheduling proposals during heatwaves, further research is required to better understand when healthcare providers are willing to shift appointments and which interventions could help promote the behavior. For example, practices could be incentivized by reimbursing losses due to the unkept original appointments.

## 5 Conclusion

This study demonstrates that substantial reductions in heat-related health risks can be achieved by adjusting the timing of healthcare appointments. Using national data from Germany, we showed that advancing or selectively rescheduling consultations to cooler hours of the day can meaningfully decrease patients' exposure to heat, particularly for elderly populations. Despite this potential, our survey results indicate that such measures are rarely practiced, depending on healthcare providers' awareness of heat-related risks. Provider education and institutional incentives for rescheduling appointments could represent effective strategies to enhance heat protection in healthcare settings as heatwaves become more frequent and intense.

## CRediT authorship contribution statement

**Philipp Sprengholz:** Writing – original draft, Methodology, Investigation, Formal analysis, Conceptualization. **Robert W. Bruckmann:** Writing – review & editing, Methodology, Investigation, Formal analysis, Conceptualization.

## Funding

This work was supported by the German 10.13039/501100003107Federal Ministry of Health (Project HEATCOM, funding number 2523FSB10B).

## Declaration of competing interest

The authors declare that they have no known competing financial interests or personal relationships that could have appeared to influence the work reported in this paper.

## Data Availability

Data will be made available on request.

## References

[bb0005] Anderson G.B., Bell M.L., Peng R.D. (2013). Methods to calculate the heat index as an exposure metric in environmental Health Research. Environ. Health Perspect..

[bb0010] Ballester J., Quijal-Zamorano M., Méndez Turrubiates R.F., Pegenaute F., Herrmann F.R., Robine J.M., Basagaña X., Tonne C., Antó J.M., Achebak H. (2023). Heat-related mortality in Europe during the summer of 2022. Nat. Med..

[bb0015] ECMWF (2024).

[bb0020] Faul F., Erdfelder E., Buchner A., Lang A.-G. (2009). Statistical power analyses using G*power 3.1: tests for correlation and regression analyses. Behav. Res. Methods.

[bb0025] Gallo E., Quijal-Zamorano M., Méndez Turrubiates R.F., Tonne C., Basagaña X., Achebak H., Ballester J. (2024). Heat-related mortality in Europe during 2023 and the role of adaptation in protecting health. Nat. Med..

[bb0030] Hansen A., Bi P., Nitschke M., Pisaniello D., Newbury J., Kitson A. (2011). Older persons and heat-susceptibility: the role of health promotion in a changing climate. Health Promot. J. Austr..

[bb0035] Jay O., Capon A., Berry P., Broderick C., De Dear R., Havenith G., Honda Y., Kovats R.S., Ma W., Malik A., Morris N.B., Nybo L., Seneviratne S.I., Vanos J., Ebi K.L. (2021). Reducing the health effects of hot weather and heat extremes: from personal cooling strategies to green cities. Lancet.

[bb0040] Kenney W.L., Chiu P. (2001). Influence of age on thirst and fluid intake. Med. Sci. Sports Exerc..

[bb0045] Kenny G.P., Tetzlaff E.J., Journeay W.S., Henderson S.B., O’Connor F.K. (2024). Indoor overheating: a review of vulnerabilities, causes, and strategies to prevent adverse human health outcomes during extreme heat events. Temperature.

[bb0050] Lu Y.-C., Romps D.M. (2023). Predicting fatal heat and humidity using the heat index model. J. Appl. Physiol..

[bb0055] National Weather Service (2022). The heat index equation. https://www.wpc.ncep.noaa.gov/html/heatindex_equation.shtml.

[bb0060] Nielsen J.B., Andersen H.S. (2025).

[bb0065] Oberai M., Baker S., Bach A.J.E., Forbes C., Jackman E., Binnewies S., Xu Z., Cunningham S., Nghiem S., Phung D., Rutherford S. (2024). Towards improvement of heatwave warnings for older adults: the case of Queensland Australia. J. Prim. Care Community Health.

[bb0070] Perkins-Kirkpatrick S.E., Lewis S.C. (2020). Increasing trends in regional heatwaves. Nat. Commun..

[bb0075] Wee J., Tan X.R., Gunther S.H., Ihsan M., Leow M.K.S., Tan D.S.-Y., Eriksson J.G., Lee J.K.W. (2023). Effects of medications on heat loss capacity in chronic disease patients: health implications amidst global warming. Pharmacol. Rev..

